# Correlation between the thyroid hormone levels and nonalcoholic fatty liver disease in type 2 diabetic patients with normal thyroid function

**DOI:** 10.1186/s12902-022-01050-2

**Published:** 2022-05-31

**Authors:** Yuanyuan Zhang, Juyi Li, Huaizhen Liu

**Affiliations:** Department of Endocrinology, Geriatrics Center, The First Affiliated Hospital of Anhui University of Traditional Chinese Medicine, Hefei, 230001 Anhui China

**Keywords:** Type 2 diabetes mellitus, Nonalcoholic fatty liver disease, Thyroid hormones, Progressive liver fibrosis

## Abstract

**Background:**

The objective of this study is to retrospectively analyze the correlation between the thyroid hormones and nonalcoholic fatty liver disease (NAFLD) in type 2 diabetes mellitus (T2DM) patients with normal thyroid function. Methods: Totally 586 T2DM patients with normal thyroid function participated in this research and were divided into T2DM without NAFLD (240 cases) group and T2DM with NAFLD (346 cases) group. The NAFLD fibrosis score (NFS) > 0.676 was defined as progressive liver fibrosis and used to categorize the patients into T2DM without progressive liver fibrosis group (493 cases) and T2DM with progressive liver fibrosis group (93 cases). Results: The results indicated that the levels of free triiodothyronine (FT3), total triiodomethylamine (TT3) and FT3/free thyroxine ratio (FT3/FT4) were significantly higher while the FT4 level was lower in T2DM with NAFLD group than that in T2DM without NAFLD group (*p < 0.05*). The levels of FT3, FT4, TT3 and TT4 in patients with progressive liver fibrosis were significantly lower in patients with progressive liver fibrosis than that in patients without progressive liver fibrosis (*p < 0.05*). Logistic regression analysis showed a positive connection between FT3/FT4 ratio and NAFLD (*p = 0.038*), a negative relationship between FT4 level and NAFLD (*p = 0.026*), between the levels of FT4, TT3 and total thyroxine (TT4) and the risk of progressive hepatic fibrosis (*p = 0.022, p = 0.007, p = 0.046*).

**Conclusion:**

There is a certain correlation between thyroid hormone levels and NAFLD in T2DM patients, suggesting that the assessment of thyroid hormone levels in T2DM patients with normal thyroid function could be helpful in the prevention and treatment of NAFLD.

## Background

Nonalcoholic fatty liver disease (NAFLD) is a clinical pathological syndrome and one of its key characteristics is hepatic steatosis which can be caused by excessive accumulation of fat in the liver [1]. The disease spectrum includes nonalcoholic steatosis (NAS), nonalcoholic steatohepatitis (NASH), liver fibrosis and liver cancer [2]. A meta-analysis of 35,599 patients with type 2 diabetes mellitus (T2DM) from 6 countries indicated that the prevalence of NAFLD was 59.67% [3]. NAFLD can significantly increase the prevalence of chronic complications in patients with T2DM, on the other hand, T2DM can stimulate the development from NAFLD to NASH and make it easy to progress to liver fibrosis [4].

Nowadays, liver biopsy is the “gold standard” in diagnosing NAFLD and progressive liver disease. However, liver biopsy is an invasive approach, so Angulo et al. [5] recommended the use of NAFLD liver fibrosis score (NFS) as preliminary evaluation of liver fibrosis. NFS is a non-traumatic system scoring liver fibrosis and NFS > 0.676 is usually used as marker of progressive liver fibrosis. These patients with NFS > 0.676 are prone to progression of cirrhosis or even liver cancer while NFS < -1.455 is the exception to progressive liver fibrosis. However, NFS is rarely investigated in T2DM patients, and most studies were based on the use of color ultrasound which can only identify NAFLD with liver fat content greater than 30% [6]. Zhang et al. [7] pointed out that the liver fat content of T2DM patients decreased while the liver fibrosis score was higher in these patients, suggesting that the use of liver color ultrasound diagnosis may underestimate the occurrence of NAFLD. Therefore, it is necessary to find other strategy for effective assessment of NAFLD in T2DM patients.

The liver plays a crucial role in the metabolism of cholesterol and triglycerides (TG). Meanwhile, thyroid hormones interact on hepatic lipid homeostasis through multiple pathways, including stimulation of free fatty acid delivery to the liver for reesterification to TG, and increasing fatty acid β-oxidation, thereby affecting hepatic fat accumulation [8]. Lower thyroid hormone level can increase blood lipids and increase the prevalence of NAFLD. Byrne et al. [9] pointed out that hypothyroidism was a key element for the occurrence of NAFLD. In contrary, Lee et al. [10] pointed out that hypothyroidism was not correlated with the occurrence of NAFLD. Van den Berg et al. [11] studied people with normal thyroid function and concluded that the free triiodothyronine (FT3) of NAFLD patients was very high, and free thyroxine (FT4) was very low. Kim et al. [12] found subclinical hypothyroidism was one of the independent factors for progressive liver fibrosis. A recent study indicated an association between thyroid hormone and liver level of triglyceride in T2DM patients [13]. These studies suggested the necessity to clarify the relationship between thyroid hormones with NAFLD, particularly for these T2DM patients.

## Methods

### Research subjects

This study is a retrospective clinical analysis of 980 T2DM patients recruited and admitted, from March 2016 to December 2018, to the department of endocrinology of the First Affiliated Hospital of Anhui University of Traditional Chinese Medicine. The diagnosis of T2DM followed the criteria proposed by the WHO Diabetes Expert Committee in 1999 and normal thyroid function was defined as the levels of thyroid hormones in the reference range, i.e., thyroid-stimulating hormone (TSH) from 0.35 to 4.94 uIU/L, FT4 from 9.1 to 19.05 pmol/L and FT3 from 2.63 to 5.7 pmol/L. Exclusion criteria included excessive drinking (*n* = 98), malignant tumor (*n* = 56), pregnancy (*n* = 22), acute complications of diabetes (*n* = 52), acute cardiovascular events (*n* = 6), severe liver and kidney dysfunction (*n* = 89), thyroid dysfunction (*n* = 60), and other acute or chronic liver diseases (*n* = 11). Finally, 586 T2DM patients (263 men and 323 women) were included in the study. The study was approved by the ethics committee of the First Affiliated Hospital of Anhui University of Traditional Chinese Medicine and the informed consent was also signed by all participants in this study (NO.2019MCZQ02).

### General clinical information and laboratory test indicators

The general information included gender, age, height, weight, diabetes course, duration of diabetes treatment, history of drinking, past medical history (hypertension, coronary heart disease, tumor, liver disease, etc), waistline, hipline, systolic pressure (SBP) and diastolic pressure (DBP). All patients were collected samples of venous blood on an empty stomach in the early morning on the second day after admission. The whole blood was centrifuged using a centrifuge. After separation of serum, fasting blood glucose (FBG), blood fat, liver function, kidney function and albumin (ALB) were measured using an automatic biochemical analyzer (Hitachi, 7600–020). Fasting C-peptide (FCP) was detected using enzyme-linked immunosorbent assay (Autolumo A2000 Plus). High-performance liquid chromatography was performed for the determination of glycosylated hemoglobin (HbA1c) and flow cytometry (Sysmex XN9000) for the determination of platelet (PLT). Chemiluminescence microparticle immunoassay (Abbott ARCHITECT i4000) was used to measure FT3, FT4, TT3, TT4 and TSH. All methods were performed in accordance with the relevant guidelines and regulations of the lab.

### Definition, calculation and group

NAFLD was diagnosed with abdominal color ultrasound by a senior physician in the hospital. The imaging diagnosis of fatty liver requires to meet the following ultrasound findings: high echo in the proximal diffusing point of the liver, higher echo intensity in the liver than in the kidney and unclear intrahepatic tube structure. Under this situation, the distant echo of liver intends to become weaker and weaker. The NAFLD diagnosis complied with the guidelines for NAFLD in China (2010 revision) [14] which requires no history of drinking. In addition to some key acute and chronic liver diseases, unexplainable continuous increase of serum indices of liver function for more than 6 months was also a requirement for diagnosis of NAFLD.

The body mass index (BMI) was calculated using the body weight (kg) and the square of the height (m^2^). Waist to hip ratio was waistline (cm)/hipline (cm). The value of modified homeostasis model assessment for insulin resistance (C-peptide) (Homa-IR (CP) was calculated by FCP instead of fasting insulin: Homa-IR (CP) = 1.5+.

FBG (mmol/L) x FCP (pmol/L)/2800 [15]. Fatty Liver-index (FLI) was calculated according to the formula published by Bedogni. FLI = (e^0.953*loge (triglycerides) + 0.139*BMI + 0.718*loge (Glutamyltransferase) + 0.053*waist circumference-15.745^)/(1 + e^0.953*loge (triglycerides) + 0.139*BMI + 0.718*loge (Glutamyltransferase) + 0.053*waist circumference-15.745^)*100 [16]. NFS was defined as NFS = (− 1.675 + 0.037 × age (years) + 0.094 × BMI (kg/m^2^) + 1.13 × impaired fasting glucose/presence of diabetes (yes = 1, no = 0) + 0.99 × asparticacid aminotransferase (AST) /alanine aminotransferase (ALT) ratio-0.013 × PLT count (× 109/^L^)-0.66 × ALB (g/dL).

According to abdominal color ultrasonography, the patients were divided into two different groups: the first group contained 240 cases of T2DM patients without NAFLD and the second group contained 346 cases of T2DM patients with NAFLD. Patient with NFS > 0.676 was defined as progressive liver fibrosis and 493 patients were classified as group of T2DM without progression liver fibrosis and 93 patients were classified as group of T2DM with progressive liver fibrosis.

### Statistical analysis

The data of the normal distribution was represented by mean ± SD. SPSS21.0 statistical software was used for the data analysis and the Kologorov-Smirnov normality of all data was tested. Comparisons were conducted within different groups with independent T-test. Measurement data for non-normal distributions were expressed as medians (interquartile intervals). Under this situation, two groups were compared using the Mann-Whitney rank sum test. The Cartridge Test was adopted to demonstrate the differences within two or more groups. The links between thyroid hormone and NAFLD were analyzed by logistic regression. *P* < 0.05 was set as the level for significant difference.

## Result

### Correlation of NAFLD with clinical data and thyroid hormone levels in T2DM patients

The clinical data and thyroid hormone levels in the participants were listed in Table 1. NAFLD was diagnosed by abdominal ultrasonography in 346 cases (59%). Patients in T2DM with NAFLD group were younger, shorter diabetes course and duration of treatment (all *p < 0.05*) when compared with patients in T2DM without NAFLD. BMI, diastolic pressure, waistline, hipline, waist hip ratio, ALT, AST, glutamyltransferase (GGT), FBG, TG, lipoprotein-B (APO-B), HOMA-IR (CP), FCP and FLI values were all significantly higher in T2DM with NAFLD group (all *p < 0.05*) when compared to T2DM without NAFLD group. FT3, TT3 and FT3/FT4 ratio were significantly elevated (*p < 0.05*) while AST/ALT, high density lipoprotein (HDL), lipoprotein-A1 (APO-A1) and FT4 levels were significantly lower in T2DM with NAFLD group than T2DM without NAFLD group (*p < 0.05*, Table 1). These results suggested that the level of thyroid hormone was associated with the morbidity risk of NAFLD in T2DM patients.

**Table 1:** Comparison of clinical data and thyroid hormone levels in T2DM patients with or without NAFLD

	T2DM without NAFLD (*n* = 240)	T2DM with NAFLD (*n* = 346)	*P* value
Sex (male/female)	101/139	162/184	0.257
Age (years)	62(54–70)	58.5(52–66)	0.002
Diabetes course (years)	10(5–14.75)	7(3–12)	0.004
Duration of treatment (years)	9(3–14)	7(1–12)	0.013
BMI (kg/m^2^)	23.81 ± 3.20	26.25 ± 3.37	0.000
Systolic pressure(mmHg)	132(122–142)	133(124–148)	0.280
Diastolic pressure(mmHg)	80(74–86)	82(76–90)	0.001
Waistline(cm)	88.22 ± 10.51	94.46 ± 9.43	0.000
Hipline (cm)	97(92–102)	101(96–105)	0.000
Waist hip ratio (WHR)	0.91(0.87–0.96)	0.93(0.90–0.97)	0.000
NFS	− 0.2479 ± 1.0663	−0.4283 ± 1.0132	0.038
ALT(U/L)	16(12–21)	20(15–30)	0.000
AST(U/L)	16(14–19)	17(15–22)	0.000
AST/ALT	1.00(0.82–1.25)	0.85(0.68–1.01)	0.000
γ- GGT (U/L)	18(14–26)	26(19–39)	0.000
AKP(U/L)	91.00(74.00–113.00)	92.00(75.75–114.00)	0.291
FBG (mmol/L)	7.11(5.75–10.41)	7.70(6.42–9.86)	0.032
TG (mmol/L)	1.16(0.84–1.64)	1.70(1.26–2.50)	0.000
TC (mmol/L)	4.51 ± 0.99	4.64 ± 0.98	0.107
HDL (mmol/L)	1.19(1.01–1.48)	1.07(0.92–1.31)	0.000
LDL (mmol/L)	2.65(2.10–3.32)	2.78(2.22–3.32)	0.276
APO-A1(g/L)	1.30 ± 0.25	1.25 ± 0.25	0.025
APO-B(g/L)	0.87 ± 0.23	0.94 ± 0.22	0.000
FT3(pmol/L)	3.95 ± 0.51	4.04 ± 0.47	0.021
FT4(pmol/L)	13.39 ± 1.53	13.04 ± 1.49	0.007
TT3(nmol/L)	1.35(1.21–1.52)	1.39(1.26–1.55)	0.032
TT4(nmol/L)	87.11 ± 15.96	85.84 ± 15.77	0.341
FT3/FT4	0.297 ± 0.044	.313 ± 0.045	0.000
TSH (uIU/L)	2.0201(1.4275–2.9372)	2.0534(1.3637–2.8333)	0.708
HOMA-IR (CP)	2.8429(2.2891–3.4687)	3.4185(2.7337–4.3036)	0.000
FCP (ng/mL)	1.47(0.99–2.03)	2.15(1.43–2.77)	0.000
HBA1C (%)	7.70(6.73–9.90)	8.00(6.80–9.63)	0.503
FLI	23.31(10.51–41.11)	53.35(34.53–71.37)	0.000

The measured data of the normal distribution was represented by mean ± SD. Measurement data for non-normal distributions were expressed as medians (interquartile intervals) *p < 0.05, **p < 0.01, ***p < 0.005 T2DM with NAFLD vs T2DM without NAFLD

*NAFLD* Nonalcoholic fatty liver disease, *T2DM* Type 2 diabetes mellitus, *BMI* Body mass index, *NFS* NAFLD fibrosis score, *ALT* Alanine aminotransferase, *AST* Aspartic acid aminotransferase, *GGT* Glutamyltransferase, *AKP* Alkaline phosphatase, *FBG* Fasting blood glucose, *TG* Triglyceride, *TC* Total cholesterol, *HDL* High density lipoprotein, *LDL* Low density lipoprotein, *APO-A1* Lipoprotein-A1, *APO-B* Lipoprotein-B, *FT3* Free triiodothyronine, *FT4* Free thyroxine, *TT3* Total triiodomethylamine, *TT4* Total thyroxine, *TSH* Thyroid stimulating hormone, *Homa-IR (CP)* Homa-Insulin resistance(C-peptide), *FCP* Fasting C-peptide, *HbA1c* Glycosylatedhemoglobin, *FLI* Fatty Liver-index. Abbreviations for other Tables are same

### Correlation of NFS with clinical information and thyroid level in T2DM patients

According to the criteria of NFS > 0.676, 93 patients (15.9%) were determined to have progressive liver fibrosis (Table 2). Patients with progressive liver fibrosis were older, with longer diabetes course and duration of treatment (*p* < 0.05) when compared to patients without progressive liver fibrosis. Diastolic blood pressure, ALT, GGT, FBG, total cholesterol (TC), low density lipoprotein (LDL) and APO-B were all significantly lower in T2DM with progressive liver fibrosis group; while BMI, waistline, hipline, waist-to-hip ratio and AST/ALT values were significantly higher (all *p* < 0.05) when compared to T2DM without progressive liver fibrosis. The levels of FT3, FT4, TT3 and TT4 were significantly lower while the level of TSH was significantly higher in T2DM with progressive liver fibrosis group when compared to T2DM without progressive liver fibrosis group (*p* < 0.05). With regard to FT3/FT4 ratio comparison, there was meaningless between two groups.

**Table 2:** Comparison of the clinical data and thyroid hormone levels in T2DM patients with or without progressive liver fibrosis

	T2DM with progressive liver fibrosis	T2DM without progressive liver fibrosis	*p* value
	NFS > 0.676(*n* = 93)	NFS < 0.676(*n* = 493)	
Sex (male/female)	34/59	229/264	0.079
Age (years)	71(66–78)	57(52–65)	0.000
Diabetes course (years)	10(6–19.5)	7(3–12)	0.000
Duration of treatment (years)	10(5–18.5)	6(1.75–12)	0.000
BMI (kg/m^2^)	26.79 ± 3.87	24.96 ± 3.36	0.000
Systolic pressure(mmHg)	135(124–149)	132(122–144.5)	0.214
Diastolic pressure(mmHg)	78(70.5–84)	82(76–90)	0.000
Waistline(cm)	96.04 ± 12.13	91.12 ± 9.79	0.000
Hipline (cm)	102(96–106.5)	99(94–103)	0.001
Waist hip ratio (WHR)	0.94(0.90–0.98)	0.92(0.89–0.96)	0.026
ALT(U/L)	15(11–20)	19(14–28)	0.000
AST(U/L)	17(15–20)	17(14–21)	0.575
AST/ALT	1.13(0.98–1.35)	0.86(0.70–1.07)	0.000
γ- GGT (U/L)	18(14–27.5)	24(17–34)	0.001
AKP(U/L)	90.0(73.0–110.5)	92.0(75.0–113.0)	0.477
FBG (mmol/L)	6.99(5.59–8.85)	7.70(6.22–10.23)	0.007
TG (mmol/L)	1.38(0.97–1.89)	1.50(1.02–2.24)	0.087
TC (mmol/L)	4.31 ± 1.08	4.64 ± 0.96	0.003
HDL (mmol/L)	1.15(0.94–1.37)	1.12(0.94–1.37)	0.971
LDL (mmol/L)	2.41(2.03–3.14)	2.80(2.24–3.36)	0.003
APO-A1(g/L)	1.26 ± 0.26	1.28 ± 0.25	0.451
APO-B(g/L)	0.85 ± 0.26	0.92 ± 0.22	0.003
FT3(pmol/L)	3.88 ± 0.53	4.03 ± 0.48	0.007
FT4(pmol/L)	12.79 ± 1.50	13.26 ± 1.50	0.006
TT3(nmol/L)	1.33(1.16–1.43)	1.39(1.25–1.55)	0.001
TT4(nmol/L)	83.36 ± 12.57	86.93 ± 16.34	0.018
FT3/FT4	0.306 ± 0.050	.307 ± 0.044	0.958
TSH (uIU/L)	2.2219(1.6204–3.2512)	2.0229(1.3759–2.8217)	0.028
HOMA-IR (CP)	3.1409(2.4986–3.8983)	3.2306(2.5629–3.9423)	0.441
FCP (ng/mL)	1.82(1.28–2.59)	1.79(1.17–2.58)	0.650
HbA1C (%)	7.60(6.80–8.85)	8.00(6.80–9.90)	0.173
FLI	40.30(19.81–61.26)	45.65(26.82–66.58)	0.090

The measured data of the normal distribution was represented by mean ± SD. Measurement data for non-normal distributions were expressed as medians (interquartile intervals) *p < 0.05, **p < 0.01, ***p < 0.005 T2DM with NFS > 0.676 vs T2DM without NFS > 0.676

*T2DM* Type 2 diabetes mellitus, *BMI* Body mass index, *NFS* Nonalcoholic fatty liver disease fibrosis score, *ALT* Alanine aminotransferase, *AST* Aspartic acid aminotransferase, *GGT* Glutamyltransferase, *AKP* Alkaline phosphatase, *FBG* Fasting blood glucose, *TG* Triglyceride, *TC* Total cholesterol, *HDL* High density lipoprotein, *LDL* Low density lipoprotein, *APO-A1* Lipoprotein-A1, *APO-B* Lipoprotein-B, *FT3* Free triiodothyronine, *FT4* Free thyroxine, *TT3* Total triiodomethylamine, *TT4* Total thyroxine, *TSH* Thyroid stimulating hormone, *Homa-IR (CP)* Homa-Insulin resistance(C-peptide), *FCP* Fasting C-peptide, *HbA1c* Glycosylatedhemoglobin, *FLI* Fatty Liver-index. Abbreviations for other Tables are same

Further analysis indicated that, for these patients with progressive liver fibrosis and NFS > 0.676, FT3/FT4 ratio were significantly higher in T2DM with NAFLD group when compared to T2DM without NAFLD group (Table 3, *p* < 0.05), However, there was no significant difference in FT3, FT4, TT3, TT4 or TSH levels between these T2DM with or without the NAFLD group (Table 3, all *p > 0.05*). Among these people without progressive liver fibrosis but with NFS < 0.676, significant differences in age, FT4 and FT3/FT4 ratio were found among T2DM patients with and without NAFLD group (Table 3, *p < 0.05*). However, there is no obvious difference in the FT3, TT3, TT4 and TSH levels between these two different groups (Table 3, *p > 0.05*).

**Table 3:** Comparison of thyroid hormone levels in T2DM patients with or without NAFLD based on the NFS level

	T2DM with progressive liver fibrosis		*p* value	T2DM without progressive liver fibrosis		*p* value
	NFS > 0.676(*n* = 93)			NFS < 0.676(493)		
	without NAFLD (*n* = 45)	with NAFLD (*n* = 48)		without NAFLD (*n* = 195)	with NAFLD (*n* = 298)	
Sex (male/female)	13/32	21/27	0.137	88/107	141/157	0.634
Age (years)	72.27 ± 8.14	69.92 ± 8.92	0.189	60(53–66)	56(51–64)	0.025
FT3(pmol/L)	3.81 ± 0.56	3.95 ± 0.50	0.199	3.98 ± 0.50	4.06 ± 0.47	0.081
FT4(pmol/L)	13.04 ± 1.57	12.55 ± 1.40	0.113	13.47 ± 1.51	13.12 ± 2.49	0.013
TT3(nmol/L)	1.29 ± 0.20	1.36 ± 0.20	0.067	1.40 ± 0.22	1.42 ± 0.23	0.215
TT4(nmol/L)	83.67 ± 11.55	83.08 ± 13.58	0.822	85.05(75.20–97.68)	84.61(73.27–95.96)	0.342
TSH (uIU/L)	2.3177 ± 0.9937	2.5104 ± 1.1098	0.381	2.0187(1.4134–2.8459)	2.0308(1.3439–2.7457)	0.537
FT3/FT4	0.295 ± 0.052	0.317 ± 0.045	0.032	0.298 ± 0.042	0.312 ± 0.045	0.000

The measured data of the normal distribution was represented by mean ± SD. Measurement data for non-normal distributions were expressed as medians (interquartile intervals) *p < 0.05,**p < 0.01, ***p < 0.005,with NAFLD vs without NAFLD

*NAFLD* Nonalcoholic fatty liver disease, *T2DM* Type 2 diabetes mellitus, *BMI* Body mass index, *NFS* NAFLD fibrosis score, *FT3* Free triiodothyronine, *FT4* Free thyroxine, *TT3* Total triiodomethylamine, *TT4* Total thyroxine, *TSH* Thyroid stimulating hormone

### Correlation of thyroid hormone levels with liver fibrosis and NAFLD in T2DM patients

The level of FT3, FT4, TT3 and FT3/FT4 ratio was specified as independent variables with different regression models, respectively, and regression models 1, 2 and 3 were constructed, regardless of whether NAFLD was a dependent variable or not. Model 1 did not correct any factors, according to the results (Table 4), suggested that the relationship between FT3,TT3 and FT3/FT4 ratio and NAFLD (*p* = 0.022, *p* = 0.043, *p* = 0.000) was considered to be positive, but the correlation between FT4 level and NAFLD was in contrary (*p* = 0.007). Model 2 corrected the gender factor and claimed that FT3, FT3/FT4 ratio were positively correlated with NAFLD (*p* = 0.034, *p = 0.000).* Meanwhile, a negative relationship could be found between the FT4 level and NAFLD (*p* = 0.006). TT3 level was not a favorable correlation with NAFLD (*p = 0.055*). Model 3 corrected for gender, age, DBP, waistline, diabetes course, BMI, TG, HDL, APO-A1 and APO-B factors. Based on the research results, it was easy to find that FT3/FT4 ratio was positively connected to NAFLD (*p = 0.038*), while FT4 level was negatively related to NAFLD (*p = 0.026*).

**Table 4:** Multiple factors logistic regression analysis of thyroid hormones and risk of NAFLD and progressive liver fibrosis

	Thyroid hormones and the risk of NAFLD			Thyroid hormones and the risk of progressive liver fibrosis			when NFS < 0.676, FT4 and the risk of NAFLD		
	OR	95%CI	*p* value	OR	95%CI	*p* value	OR	95%CI	*p* value
FT3									
Model 1	1.489	1.059–2.094	0.022*	0.532	0.334–0.846	0.008**			
Model 2	1.456	1.029–2.058	0.034*	0.56	0.35–0.899	0.016*			
Model 3	1.041	0.7–1.549	0.842						
Model 4				0.654	0.363–1.176	0.156			
FT4									
Model 1	0.86	0.77–0.96	0.007**	0.798	0.679–0.937	0.006**	0.859	0.761–0.969	0.014*
Model 2	0.857	0.767–0.957	0.006**	0.802	0.683–0.942	0.007**			
Model 3	0.865	0.762–0.982	0.026*						
Model 4				0.796	0.655–0.967	0.022*			
Model 5							0.856	0.758–0.968	0.013*
Model 6							0.801	0.672–0.955	0.013*
TT3									
Model 1	2.171	1.025–4.602	0.043*	0.142	0.047–0.431	0.001***			
Model 2	2.095	0.985–4.457	0.055	0.152	0.05–0.464	0.001***			
Model 3	1.166	0.495–2.726	0.726						
Model 4				0.151	0.038–0.597	0.007**			
TT4									
Model 1				0.985	0.97–1.0	0.047*			
Model 2				0.984	0.969–0.999	0.033*			
Model 4				0.98	0.962–1.0	0.046*			
TSH									
Model 1				1.252	1.016–1.544	0.035*			
Model 2				1.224	0.99–1.513	0.062			
Model 4				1.13	0.884–1.446	0.33			
FT3/FT4									
Model 1	2870.6	59.9–137,433.9	0.000***				2023.8	27.3–150,045.9	0.001***
Model 2	2493.8	50.9–122,059.1	0.000***						
Model 3	99.0	1.3–7567.3	0.038*						
Model 5							1395.9	17.9–108,303.0	0.001***
Model 6							7545.9	18.4–3,093,672.6	0.004***

Annotation: Model 1 did not correct any factors. Model 2 corrected gender factor. Model 3 corrected gender, age, diastolic pressure, waistline, diabetes course, body mass index, triglyceride, high density lipoprotein, lipoprotein-A1 and lipoprotein-B factors. Model 4 corrected gender, age, diastolic pressure, waistline, diabetes course, and body mass index, total cholesterol and lipoprotein-B factors. Model 5corrected gender and age factors. Model 6 corrected gender, age, TT3, TT4, TSH factors

*NAFLD* nonalcoholic fatty liver disease, *NFS* NAFLD fibrosis score, *FT3* free triiodothyronine, *FT4* free thyroxine, *TT3* total triiodomethylamine, *TT4* total thyroxine, *TSH* thyroid stimulating hormone

^*^p < 0.05

^**^p < 0.01

^***^p < 0.005

FT3, FT4, TT3, TT4 and TSH were specified as arguments, respectively, and regression models 1, 2 and 4 were constructed regardless of whether they were dependent variables on progressive liver fibrosis (Table 4). In Model 1 which did not correct any factors, FT3, FT4, TT3 and TT4 levels were inversely connected with the risk of progressive hepatic fibrosis (*p = 0.008, p = 0.006, p = 0.001, p = 0.047* respectively). A positive relationship between TSH and the risk of progressive hepatic fibrosis (*p = 0.035*) was found. Model 2 corrected the factor of gender factors. The results showed that FT3, FT4, TT3 and TT4 levels were inversely related to progressive hepatic fibrosis risk (*p = 0.016, p = 0.007, p = 0.001, p = 0.033*) while TSH was not correlated with progressive hepatic fibrosis risk (*p = 0.062*). Model 4 corrected factors of gender, age, diastolic blood pressure, waistline, diabetes course, BMI, TC and APO-B. As shown in Table 4, the levels of FT4, TT3 and TT4 can be greatly influenced by the risk of progressive hepatic fibrosis (*p = 0.022, p = 0.007, p = 0.046*, respectively).

When NFS < 0.676, FT4 and FT3/FT4 ratio as independent variable were assigned with NAFLD as dependent variables, the regression models 1, 5 and 6 were constructed. Model 1 did not correct any factors and showed that the FT4 level had negative correlation with the risk of NAFLD (*p = 0.014*), the FT3/FT4 ratio had positive correlation with the risk of NAFLD (*p = 0.001*). Model 5 corrected gender and age factors and showed that the FT4 level had negative correlation with the risk of NAFLD (*p = 0.013*), the FT3/FT4 ratio was inversely related to the risk of NAFLD (*p = 0.001*). Model 6 corrected gender, age, TT3, TT4,TSH and showed that the FT4 levels had negative correlation with the risk of NAFLD (*p = 0.008*), while, a positive relationship between FT3/FT4 ratio and the risk of NAFLD (*p = 0.004*).

Further explore the multiple stepwise logistic regression analysis of the influence of thyroid hormone level on NAFLD and progressive liver fibrosis (Table 5). With NAFLD as dependent variable and 24 meaningful indicators in Table 1 as independent variables, meanwhile, with progressive liver fibrosis as dependent variable and 20 meaningful indicators in Table 2 as independent variables (included indicators were channeled in actual values), multiple stepwise logistic regression analysis was conducted. The results showed that diabetes course, FT4, HOMA-IR (CP), and FLI were the influencing factors of NAFLD, meanwhile, age, BMI, FT4 and HOMA-IR (CP) were the influencing factors of progressive liver fibrosis (*p < 0.05*).

**Table 5:** Stepwise multiple Logistic regression analysis of the influencing factors of NAFLD and progressive liver fibrosis in euthyroid subjects

Independent variable	Thyroid hormones and the risk of NAFLD				Thyroid hormones and the risk of progressive liver fibrosis			
	B	OR	95%CI	*p* value	B	OR	95%CI	*p* value
Age	−0.017	0.984	0.964–1.003	0.102	0.136	1.145	1.109–1.183	0.000
Diabetes course	−0.031	0.970	0.941–0.999	0.046	−0.010	0.990	0.952–1.029	0.604
BMI	−0.001	0.999	0.913–1.092	0.974	0.333	1.396	1.225–1.590	0.000
FT3	0.117	1.124	0.747–1.692	0.575	−0.331	0.719	0.398–1.296	0.272
FT4	−0.156	0.855	0.751–0.974	0.018	−0.251	0.778	0.638–0.949	0.013
HOMA-IR (CP)	0.17	1.186	1.006–1.397	0.042	−0.256	0.774	0.616–0.973	0.028
FLI	0.043	1.044	1.031–1.058	0.000	−0.017	0.984	0.9661.002	0.074

Annotation: *NAFLD* nonalcoholic fatty liver disease, *BMI* Body mass index, *FT3* free triiodothyronine, *FT4* free thyroxine, *Homa-IR (CP)* Homa-Insulin resistance(C-peptide), *FLI* Fatty Liver-index

^*^p < 0.05

^**^p < 0.01

^***^p < 0.005

**Fig. 1:**
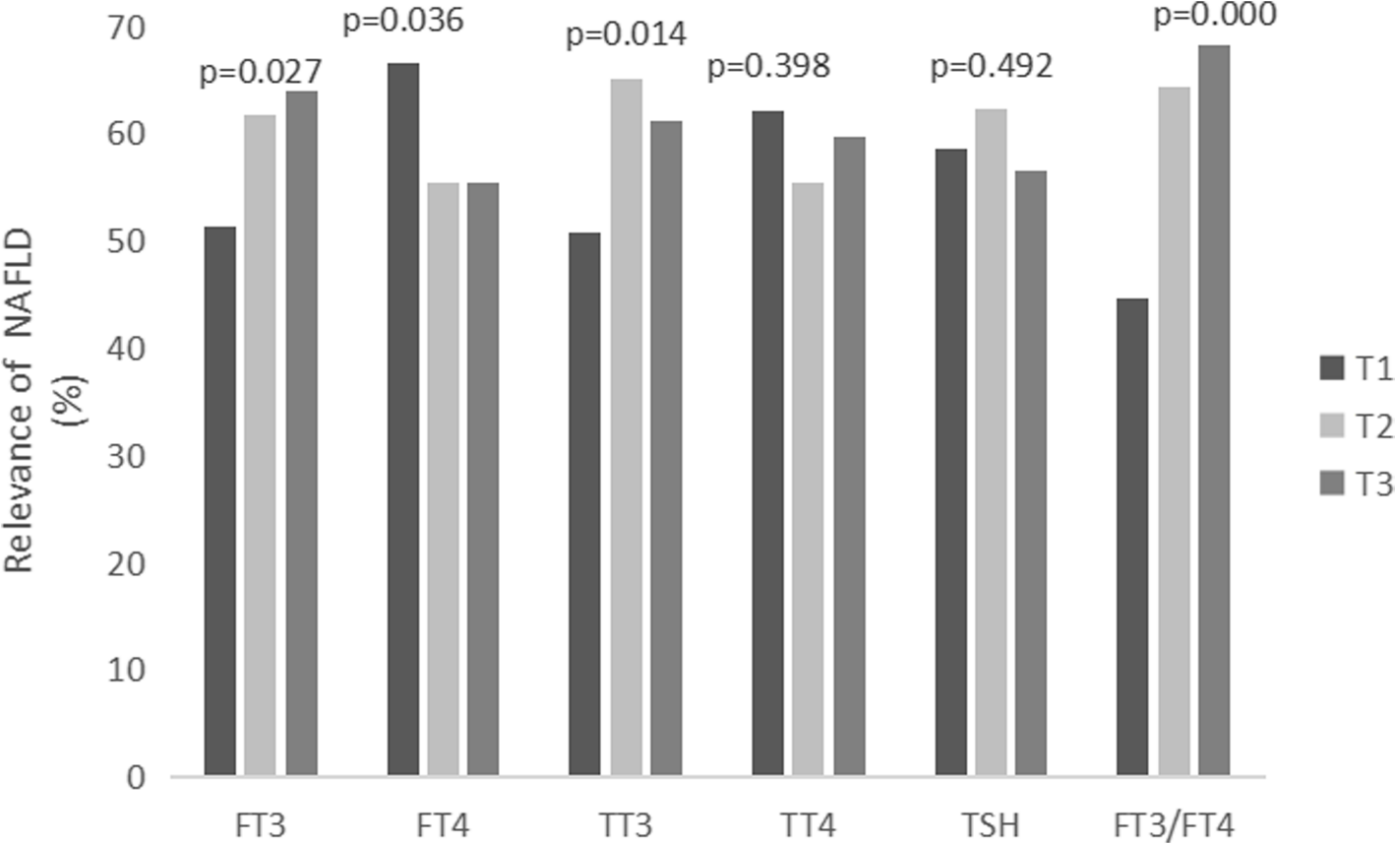
Showed the relevance of the incidence of NAFLD with thyroid hormone levels. All patients pooled together and analyzed according to the three-point numbers of FT3, FT4, TT3, TT4, FT3/FT4 ratio and TSH levels. The results suggested that the incidence of NAFLD showed a significant increase trend with levels of FT3 and FT3/FT4 ratio, increasing, however, contrary to FT4 (*p* < 0.05)

**Fig. 2:**
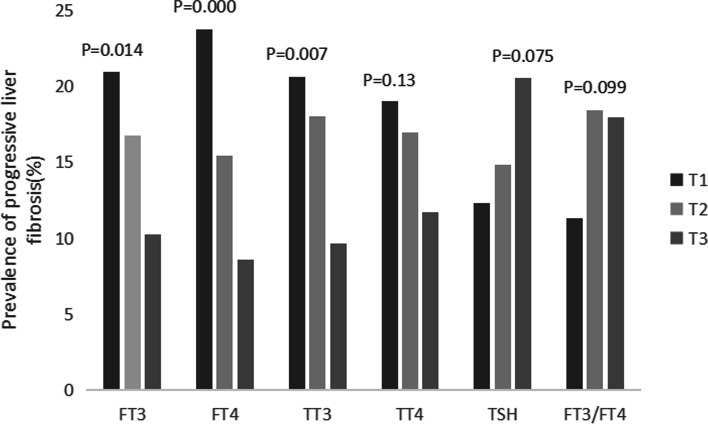
Showed the relevance of the incidence of progressive liver fibrosis with thyroid hormone levels. The incidence of progressive hepatic fibrosis displayed a decrease trend under the increased levels of FT3, FT4 and TT3 (*p* < 0.05). Annotation: NAFLD, Nonalcoholic fatty liver disease; FT3, Free triiodothyronine; FT4, Free thyroxine; TT3, Total triiodomethylamine; TT4, Total thyroxine; TSH, Thyroid stimulating hormone; T1, T2, T3, three-point numbers

Finally, all patients were pooled together and the relevance between the levels of FT3, FT4, TT3, TT4, TSH, and FT3/FT4 ratio with the incidence of NAFLD and progressive liver fibrosis was analyzed. The three-point numbers of FT3 were T1 (<3.8 pmol/L)、T2 (3.8 ~ 4.21 pmol/L) and T3 (≥4.21 pmol/L). The three-point numbers of FT4 were T1 (<12.41 pmol/L)、T2 (12.41 ~ 13.7 pmol/L) and T3 (≥13.7 pmol/L). The three-point numbers of TT3 were T1 (<1.29 nmol/L)、T2 (1.29 ~ 1.46 nmol/L) and T3 (≥1.46 nmol/L). The three-point numbers of TT4 were T1 (<77.71 nmol/L)、T2 (77.71 ~ 91.32 nmol/L) and T3 (≥91.32 nmol/L). The three-point numbers of TSH were T1 (<1.6312uIU/L)、T2 (1.6312 ~ 2.5794uIU/L) and T3 (≥2.5794uIU/L). FT3/FT4 ratio were T1 (<0.2865)、T2 (0.2865 ~ 0.3239) and T3 (≥0.3239). The results showed that the incidence of NAFLD presented a significant increase trend with levels of FT3 and FT3/FT4 ratio increasing (*p < 0.05*; Fig. 1). Following the FT4 level became higher and higher, the NAFLD showed an obvious decrease trend. When the TT3 level progressed from<1.29 to 1.29 ~ 1.46, the incidence of NAFLD showed an increase trend (*p < 0.05*, Fig. 1). There was no relevance between the levels of TT4 and TSH and the incidence of NAFLD (*P > 0.05*, Fig. 1). For the relevance between the levels of thyroid hormones and the incidence of progressive hepatic fibrosis, the results showed a decrease trend with the increase of FT3, FT4 and TT3 levels (*p < 0.05*, Fig. 2). There was no relevance between the levels of TT4, FT3/FT4 and TSH and the incidence of progressive hepatic fibrosis (*P > 0.05,* Fig. 2).

### FT3, FT4, FT3/FT4 predicted the risk of NAFLD

ROC curve was used to evaluate the influence of FT3, FT4 and FT3/FT4 ratio on predictive efficacy of NAFLD risk. The results showed that in T2DM patients, FT3, FT4 and FT3/FT4 ratio could effectively predict the risk of NAFLD (FT3: AUC = 0.553, 95% CI 0.505 ~ 0.601, *P = 0.029*; FT4: AUC = 0.564, 95% CI 0.517 ~ 0.611, *P = 0.009*; FT3/FT4 ratio: AUC = 0.600, 95% CI 0.554 ~ 0.647, *P = 0.000*, respectively). The best cut-off point was: FT3 = 3.805, the sensitivity was 71.1%, the specificity was 40.4%; FT4 = 12.67, the sensitivity was 45.1%, the specificity was 66.7%; FT3/FT4 ratio = 0.2864, the sensitivity was 74.9%, the specificity was 45%.

## Discussion

More and more studies showed that the volatility of serum level of thyroid hormones within normal range had a great chance to be associated with the risk of NAFLD [11, 17, 18]. In the present study, the level of FT3 and FT3/FT4 ratio in the NAFLD group was higher than the non-NAFLD group, and the FT4 levels were lower. After correcting the mixed factors such as diabetes course, BMI, systolic pressure, TG and HDL, the result indicated that the risk of NAFLD showed a significant increase trend with the levels of FT3 and FT3/FT4 ratio and increasing, and decline trend with the levels of FT4 increasing. Through ROC curve analysis, this study found that FT3, FT4 and FT3/FT4 ratio could be used as a serological reference index to predict the risk of NAFLD.

These studies together suggest an alternative option for diagnosis strategy of NAFLD in clinic, as liver biopsy is actually a traumatic examination that limits its clinical application although it is considered to be the “gold standard” of the diagnosing of NAFLD. It is clear that thyroid hormone is involved in human lipid metabolism, induces lipolysis in the liver and participates in the storage and degradation of lipid droplets in liver lysosome [19]. When the thyroid function is low, the liver’s lipase activity and triglyceride elimination is reduced, resulting in intrahepatic triglyceride accumulation and thereby promoting the development of NAFLD. In addition, low thyroid hormone level also affects the adipocytokines in the circulating system, such as tumor necrosis factor-alpha, leptin, adiponectin, etc. [20]. Adipocytokines promote the formation of hepatitis and liver fibrosis by direct hepatotoxicity or by promoting the formation of free radical [21]. The correlation between thyroid hormones and liver fibrosis has been verified in clinic by previous studies [12, 17, 22–24]. Liu et al. [23] discussed the results of 1773 health examinations. Liver fibrosis was assessed by BARD score ≥ 2. Under this situation, the FT3 levels independently have a close relationship with hepatic fibrosis risk which takes place among patients with NAFLD. Bano [17] participated in 9419 participants, and the liver fibrosis level was detected by using transientselastography. After correcting gender, age, taking lipid-lowering drugs, cardiovascular risk factors and follow-up time described that TSH levels and FT4 levels were positively linked with liver fibrosis risk and risk of liver fibrosis correspondingly.

However, it should be pointed out that these clinical research subjects from above previous studies were all non-diabetic subjects. Currently, it has been clarified that diabetic patients are often accompanied by abnormal level of thyroid hormones. Higher incidence of NAFLD has been confirmed in T2DM patients and lower screening threshold has been proposed for clinicians [25, 26]. Bril et al. [13] demonstrated that low levels of FT4 increased triglyceride levels in T2DM patients followed the increasing risk of NAFLD by taking a study on 232 patients with normal thyroid function in T2DM, which was consistent with the present result that an obvious increase trend of NAFLD incidence was accompanied by the declining of FT4 levels. Zhu et al. [27] used the MatchIt function in the R language package to study 184 T2DM patients using 1:1 covariation matching. Here, the level of FT3, TT3 and FT3/FT4 ratio in the NAFLD group was higher than the non-NAFLD group. After correcting the mixed factors such as diabetes course, BMI, systolic pressure, TG, HDL and LDL, the result found that the serum FT3 levels had a positive relationship with NAFLD’s risk. Meanwhile, the results of NAFLD showed a significant upward trend with the levels of FT3 and FT3/FT4 ratio increasing, which were consistent with our result in the present study. And in our research, when NFS < 0.676, significant differences in age, FT4 and FT3/FT4 ratio were found among T2DM patients with and without NAFLD group. These results are consistent with the results of previous study by Van den berg [11], suggesting that abnormal thyroid hormone level is one valuable indicator worthy of emphasis for diagnosis of T2DM. The mechanism for the phenomenon may be that metabolic disorder induced by abnormal thyroid hormone functions further increases the cellular resistance to insulin [28].

As for the relationship between TSH and NAFLD, many studies have proved that subclinical hypothyroidism is a risk factor for NAFLD, and the increase of TSH level is positively correlated with the incidence of NAFLD [29, 30]. However, the research conclusion for the population with normal thyroid function was not consistent. Some scholars believed that if TSH was at the high limit of normal value in people with normal thyroid function, it was related to NAFLD [31]. But Ittermann et al. [32] had found that TSH level had nothing to do with hepatic steatosis in people with normal thyroid function. In this research, when diagnosing fatty liver by abdominal color doppler ultrasound, the levels of TSH in the NAFLD group was no difference with the non-NAFLD group, however, From the perspective of liver fibrosis, we found that the progressive hepatic fibrosis group had low FT3, FT4, TT3 levels and high TSH levels. Kim et al. [24] also concluded that hypothyroidism was an independent predictor of progressive hepatic fibrosis.

Unfortunately, we only found that a positive relationship between TSH and the risk of progressive hepatic fibrosis (*p = 0.035*) which did not correct any factors. While TSH was not correlated with progressive hepatic fibrosis risk which corrected the factor of other factors. There some limitations in the present study. One limitation is that the size of samples is relative small. In addition, the effects of various chronic complications such as cardiovascular and cerebrovascular diseases, hypoglycemic drugs and lipid-regulating drugs on thyroid hormone levels, differences in analytical methods were not considered, and further work is required in the future. The definition of normal thyroid function and differences in the criteria of NAFLD diagnosis might affect the inconsistency of conclusions. Therefore, a correct and thorough understanding of the results in this study requires certain consideration in clinical practice.

## Conclusion

In summary, the present study indicated that there is closely correlation between the abnormal thyroid hormone levels and liver fibrosis in T2DM patients, i.e., the prevalence of NAFLD increased following the increase of FT3, FT3/FT4 ratio and decrease of FT4. These results suggested that the change of thyroid hormone level in T2DM patients should be tested routinely for judging the patient’s condition and predicting the prognosis.

## Data Availability

The data that support this study are available from the corresponding author only upon reasonable request, once the study has been published.

## Abbreviations

NAFLD: Nonalcoholic fatty liver disease
T2DM: Type 2 diabetes mellitus
NFS: Nonalcoholic fatty liver disease fibrosis score
FT3: Free triiodothyronine
FT4: Free thyroxine
TSH: Thyroid-stimulating hormone
IR: Insulin resistance
NAS: Nonalcoholic steatosis
NASH: Non-alcoholic steatohepatitis
SBP: Systolic pressure
DBP: Diastolic pressure
FCP: Fasting C-peptide
Homa-IR (CP): Modified homeostasis model assessment for insulin resistance (C-peptide)
AST: Asparticacid aminotransferase
ALT: Alanine aminotransferase
GGT: Glutamyltransferase
AKP: Alkaline phosphatase
FBG: Fasting blood glucose
TG: Triglyceride
TC: Total cholesterol
HDL: High density lipoprotein
LDL: Low density lipoprotein
APO-A1: Lipoprotein-A1
APO-B: Lipoprotein-B
ALB: Albumin
HbA1c: Glycosylated hemoglobin
PLT: Platelet
FLI: Fatty Liver-index
SD: Standard deviation
OR: Odds ratio
CI: Confidence interval
